# A single mutation at position 214 of influenza B hemagglutinin enhances cross-neutralization

**DOI:** 10.1080/22221751.2025.2467770

**Published:** 2025-02-17

**Authors:** Ziqi Cheng, Yeqing Sun, Yanru Shen, Xi Wu, Ling Pan, Hao Wu, Yunbo Bai, Chenyan Zhao, Junfeng Ma, Weijin Huang

**Affiliations:** a National Engineering Laboratory for AIDS Vaccines, School of Life Sciences, Jilin University, Changchun, People’s Republic of China; b Division of HIV/AIDS and Sexually transmitted Virus Vaccines, Institute for Biological Product Control, National Institutes for Food and Drug Control (NIFDC) and WHO Collaborating Center for Standardization and Evaluation of Biologicals, Beijing, People’s Republic of China; c Graduate School of Peking Union Medical College, Beijing, People’s Republic of China; d School of Pharmacy, Shenyang Pharmaceutical University, Shenyang, People’s Republic of China

**Keywords:** IBV, hemagglutinin, mutation, cross-neutralization, vaccine

## Abstract

High variability of influenza B virus (IBV) hemagglutinin (HA) impairs the cross- neutralization ability of vaccines, leading to reduce efficacy. We identified significant differences in cross-neutralization between IBV strains B/Wyoming/06/2014 and B/Brisbane/60/2008, which differ in only three amino acid residues. The 214 T point mutation was found to dramatically enhance cross-neutralization (>10-fold). Antibody-based reverse validation also revealed that this mutation significantly increased the neutralization capacity (500–62,500-fold). Furthermore, monitoring revealed that the mutation rate at this site has reached its highest level in nearly 20 years, with a prevalence exceeding 80% in sequences submitted from certain regions. Our findings provide new evidence for the selection of vaccine strains with improved cross- neutralization effects, which will aid the development of broad-spectrum vaccines by modifying minimal antigenic epitopes.

## Introduction

Influenza B virus (IBV) is one of the most common pathogens affecting humans, leading to a considerable global public health burden [1]. Epidemics of IBV occur every 2–4 years, resulting in widespread absenteeism and mortality [2]. IBV is divided into the Victoria and Yamagata lineages, which generally exhibit no cross-neutralization [3,4]. However, the detection rate of the Yamagata lineage has gradually declined over the years, and the World Health Organization (WHO) advisory committee previously determined that there is no obligation to include antigens of the Yamagata lineage in influenza vaccines [5–8]. Therefore, the greatest challenge for IBV vaccines remains protection from the Victoria lineage.

Hemagglutinin (HA) is the most prominent membrane protein of the influenza virus, and eliciting neutralizing antibodies against it plays a crucial role in generating protection against influenza in humans [9]. However, due to frequent mutations in HA, vaccine strains must be continually updated in order to target the latest circulating viruses, yet mismatches still frequently occur [10,11]. Thus, enhancing the cross- neutralization capacity of vaccine strains and identifying epitopes that induce broadly neutralizing antibodies are current research priorities [12–15]. Some single-point mutations in the HA of influenza A induce flexible pivot antibodies, which can accommodate glycosylation differences in the conserved stalk domain, enabling cross-group protection [16,17]. Similarly, single-point mutations present in avian influenza HA have also been found to enhance the cross-neutralization between different lineages [18]. Studies on broadly neutralizing antibodies (bnAbs) mainly focused on the conserved epitopes in the HA stalk region [19–21]. Although the head region of HA protein may potentially elicit stronger antibody response and plays a crucial role in cellular immunity, there is a lack of evidence to clarify the mechanisms of bnAbs targeting the head region, which might be due to high variability of the head [22,23]. The residue at position 214 investigated in this study is located within the 190 loop and represents the only antigenic site among the four epitopes of the influenza virus that resides in the receptor-binding site (RBS). As mutations in this region have been shown to reduce vaccine efficacy [24,25], modifying epitopes in the head region is a promising strategy to elicit robust bnAbs responses.

We previously discovered that two mutant HA proteins from the V1A lineage, B/Wyoming/06/2014 and B/Brisbane/2008, exhibit significant differences in cross- neutralization capacity [4]. In this study, we generated single and combined mutations at different positions to identify those responsible for the observed variations of cross- neutralization capacity. We found that mutations in the RBS significantly affected cross- neutralization between antigens, consistent with previous reports [26–29]. Notably, only antibodies that recognize epitopes located on the RBS can directly inhibit viral infection of cells [20]. The receptor-binding site of the RBS region was identified based on four highly conserved residues, 95F, 158W, 191H and 202Y, which are related to the characteristics and receptor-binding properties of the RBS [30]. This provides evidence for developing therapeutic antibodies or vaccines targeting the RBS region. Our research fills a gap by showing that enhanced cross-neutralization against IBV can be achieved with minimal modifications to the original antigen, offering valuable guidance for the selection of candidate vaccine strains.

## Materials and methods

### Cells and viruses

HEK293 cells were provided by Sino Biological (China) The cells were cultured in complete medium, which is composed of Dulbecco's Modified Eagle's Medium (DMEM) supplemented with 10% Fetal Bovine Serum (FBS) and a penicillin (100 U/mL)-streptomycin (100 μg/mL) solution. The 293T-α2-6 cells (overexpressing Hst6gal1 [NM_173216.2]), used in titration or neutralization assays, were provided by VectorBuilder (China) and maintained in complete medium containing 100 μg/ml hygromycin B (Invitrogen, USA). Wild-type viruses B/Austria/1359417/2021, were provided by HuaLan Biological.

### Antibody and protein expression and purification

The coding sequences of B/Wyoming/06/2014(BW06) and B/Brisbane/60/2008(BB08) were downloaded from the GISAID database and the extracellular domains of two HA proteins were cloned into the baculovirus transfer vector pFastBac (Invitrogen, USA). The sequences were modified with an N-terminal signal peptide (SP) and a C-terminal His10-tag for purification. After infecting Hi5 cells (Invitrogen), the culture supernatant was collected two days post-infection and the proteins were purified using a Ni-affinity chromatography column (17057502; GE Healthcare, USA), according to the manufacturer’s instructions. The obtained fractions were concentrated, buffer-exchanged into PBS using dialysis method, and stored at −80°C.

To generate antibodies, the sequences of CR8033, CR9114, and CR8059 (see Supplementary Table 2) were cloned into the expression vector pSTEP2 and the plasmids were transfected into 200 mL of HEK293 cells at a density of 3 × 10⁶ cells/mL. The cells were cultured in a shaking incubator with 5% CO_2_ at 37°C and 150 rpm. On day 5, the supernatant was harvested, and the target antibodies were captured using Protein A (GE Healthcare, USA) affinity chromatography.

### Construction and production of pseudotyped HA virus mutants

Full-length codon-optimized coding sequences of BW06 and BB08 hemagglutinin (HA) were cloned into the pcDNA3.1 vector. The HA mutants were generated through site-directed mutagenesis of BW06, as described previously [31]. The HA, NA, and HAT plasmids were combined to co-transfect cells Lipofectamine 3000 (Invitrogen) at a ratio of 2:2:1. The transfected cells were then infected with the G*ΔG-VSV pseudovirus. After 24 and 48 h, the viral supernatants were collected, pooled, filtered through a 0.22 μm pore-size membrane (Pall Corporation, USA), and stored at −80°C.

### Pseudovirus titration and qPCR quantification

Pseudovirus was thawed and subjected to three-fold serial dilutions in complete medium prior to the infection of 293T-α2-6 cells. After 24 h of incubation at 37°C with 5% CO_2_, the infection was assessed using a luminescence assay (Perkin Elmer, USA). The pseudovirus titre was determined by calculating the cell culture infectious dose 50% (CCID50) using the Reed-Muench method [32].

RNA extraction was conducted following the instructions provided by the pre-packaged virus automated RNA extraction kit (A52990; MagMAX, USA) The extracted RNA was amplified using VSV-specific primers, with the forward primer 5′-TCTCGTCTGGATCAGGCGG-3′ and the reverse primer 5′-TGCTCTTCCACTCCATCCTCTTGG-3′. Gene expression levels was determined using the 7500 Real-Time PCR System (Thermo Fisher Scientific, USA), The number of viral copies is calculated based on the CT value.

### Biolayer interferometry assay

For the biolayer interferometry (BLI) assay, Neu5Aca2-6GalNAca-sp3 (GlycoNZ, New Zealand) was first diluted to 5 μg/mL in running buffer (PBST + 0.5% BSA) and immobilized on SA biosensors (SARTORIUS, Germany). Immobilization levels were set at 1.0 nm. The analytes were diluted to 50 μg/mL and then serially diluted two-fold in running buffer. Binding interactions were measured using an Octet RED384 system (SARTORIUS), with association times of 120 s and dissociation times of 180 s. Data were analyzed using GraphPad Prism 9 (GraphPad Software Inc., USA) to determine binding rates, dissociation rates, and affinity constants.

### Immunization of Guinea pigs and serum preparation

Female Hartley guinea pigs weighing 200–250 g (*n* = 5) were immunized via three intramuscular injections into the muscle. The first injection consisted of 300 μg of plasmid DNA in 500 µL ddH_2_O, while the second and third injections included 2000 pg of purified pseudovirus vaccine in 500 µL PBS. The purification and quantification of the pseudovirus were performed according to our previous methods [4]. The pcDNA3.1-derived expression vectors were subjected to both forward and reverse sequencing using the T7 promoter and bGH sequences present on the vector to ensure the fidelity of the inserted fragment. Fourteen days after the final immunization, blood samples were collected to extract the antisera. The animal study protocol was approved by the Animal Welfare and Ethics Review Committee of the National Institute for Food and Drug Control (Approval No.: NIFDC number 2024(B)018).

### Pseudovirus neutralization assay

For the pseudovirus neutralization assay, serum samples were initially diluted 1:150 in complete medium and then serially diluted three-fold. Antibody samples were diluted to 3.7 μg/mL and also serially diluted three-fold. A total of 100 TCID50 of pseudovirus in 50 μL was added to each well of a 96-well plate and incubated with the diluted serum or antibody samples for 1 h at 37°C. After incubation, 5 × 10^4^ 293T-α2-6 cells were added to each well, and the plates were incubated at 37°C with 5% CO_2_ for 24 h. Infection was determined by measuring luminescence [33], and the ID50 value (the serum dilution at which 50% neutralization is achieved) was calculated using the Reed-Muench method.

### Viral challenge experiments

100 μL of guinea pig serum was intraperitoneally injected into 6–8 week-old female BABL/c mice. Five hours later, blood was collected from the inner canthus of the eye, and an ELISA method was used to verify the success of serum transfer. Subsequently, the mice were infected with 5LD50 dose of B/Austria/1359417/2021 virus via intranasal administration. Body weight was monitored for 8 days, and euthanasia was performed when weight loss exceeded 25% of the initial body weight. The protocol of this animal study was approved by the Animal Welfare and Ethics Review Committee of the National Institute for Food and Drug Control (Approval No.: NIFDC number: 2025(B)001).

For the ELISA experiment, purified protein (2 µg/mL, 100 µL per well) was coated onto a 96-well polystyrene plate (Thermo Fisher Scientific) at 4°C overnight. The next day, the ELISA plate was washed three times with PBST, and 200 µL of blocking solution (5% BSA/PBS powder) was added to each well for a 2-hour incubation at 37°C. After removing the blocking solution, the plate was washed three times with PBST. Then, 100 µL of serially diluted mouse serum samples were added to each well and incubated at 37°C for 2 h. After incubation, the plate was washed five times with PBST, and 100 µL of HRP-conjugated anti-guinea pig antibody solution (Thermo Fisher Scientific) was added to each well. After 1 h of incubation, the antibody solution was washed five times with PBST. Then, 100 µL of TMB was added to each well and incubated for 10 min. The reaction was stopped by adding 50 µL of stop solution to each well. The absorbance at 450 nm (A450) was recorded using a microplate reader.

### Statistical analysis

Data were analyzed using GraphPad Prism 9.0 software (GraphPad Software Inc., USA) The results are presented as the means ± standard deviations (SD). Statistical significance was determined using the one-way ANOVA or Spearmanrank-correlation tests method, and significance levels were indicated with asterisks as follows: *p* < *0.05, *p* < **0.01, ***p < 0.005, and ****p < 0.001.

## Results

### Analysis of epitope differences between BW06 and BB08

**Figure 1.**
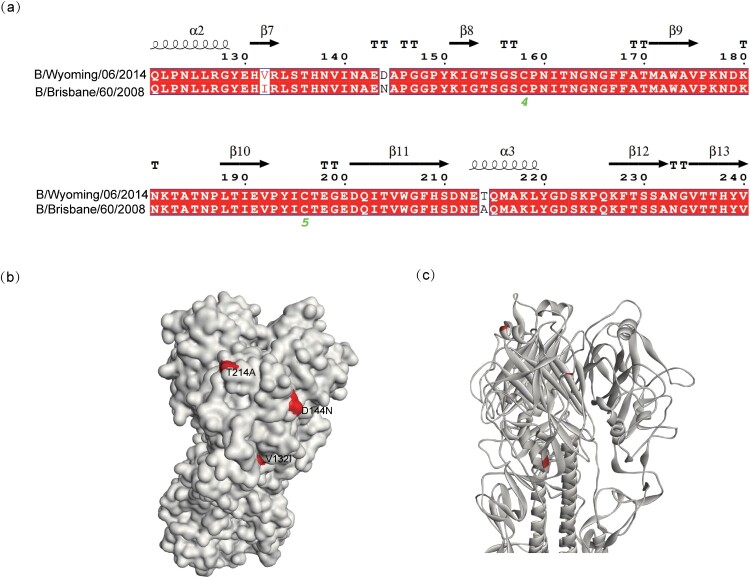
Schematic diagram of differential residues between BW06 and BB08. (a) Alignment of amino acid residues 121–240 between BW06 and BB08, with secondary structures annotated on the sequence. (b–c) 3D structure of BW06, with differential sites highlighted in red.

We previously discovered that BW06 and BB08 exhibited significant differences in their cross-neutralization capabilities [4]. Based on these findings, we aligned their secondary structures (Figure 1(a)) and found only 3 different amino acid residues between the sequences, all located in subunit HA1. To further investigate the 3D structures of the two antigens, we modelled BW06 and highlighted the positions of these differential residues in red (Figure 1(b and c)). Notably, residue 214 is located in an α-helix of the receptor-binding site. The other two residues, 132 and 144, are respectively located in a loop and a helix, within the major antigenic sites of IBV [34]. Although these two mutations occurred within the antigenic sites, both strains are classified as belonging to the V1A lineage (Supplementary Table 1).

### Differences in infectivity between BW06 and BB08

**Figure 2.**
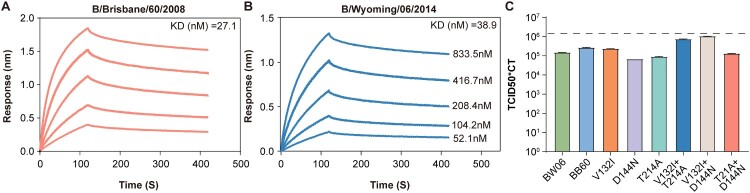
The receptor-binding affinity of BB08 and BW06 proteins and the ability of pseudoviruses expressing their mutants to infect cells. (a–b) Both proteins were diluted in a concentration gradient (833.5, 416.4, 208.4, 104.2, and 52.1 nM) and were tested for binding to glycans immobilized on the biosensor chip. The dissociation constants (Kd) for each interaction are shown in the top-right corner of the figure. (c) Viral infectivity was calculated by multiplying the TCID50 by the CT value. To ensure that the changes of CT values accurately reflect viral quantity, we applied a correction based on the principle that an increase of 1 in the CT value corresponds to a 2-fold change of quantity. Each viral group contained three replicates, and the detection threshold was set at 10 times the average value of BW06.

To assess whether the identified mutations affect the receptor-binding ability of the virus, we expressed both proteins (Supplementary Figure 1) and analyzed their binding capacity for α2-6 sialic acid. We found no significant differences in receptor-binding capacity between the two proteins (Figure 2(a and b)). However, since there is no direct correlation between receptor-binding ability and viral infectivity, we introduced point mutations from BB06 into BW06, including all combinations. All mutant proteins were packaged into pseudoviruses and quantified using qPCR. Then, 293T-α2-6 cells were infected with the pseudoviruses and the TCID50*CT value was calculated to evaluate their infectivity (Figure 2(c)). The results indicated that the pseudoviruses of different mutants all exhibited infection rates in the same order of magnitude as BW06, indicating no significant changes of infectivity (Figure 2(c)).

### Impact of single and combined mutations on the cross-neutralization ability

**Figure 3.**
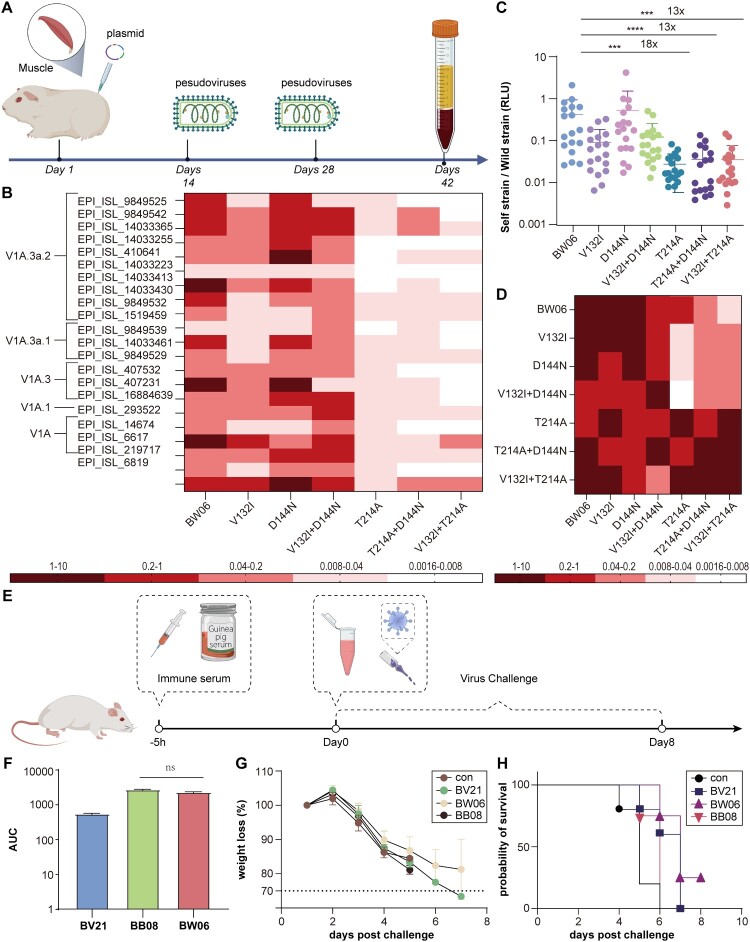
Investigating the Immunogenicity of Different Point Mutants Using a Pseudovirus Neutralization Assay. (a) Guinea pigs were given three doses of the vaccine 2 weeks apart, and serum samples were collected 14 days after the third dose. (b) The EC50 values of serum against both wild-type and mutant strains were calculated and analyzed. The ratio was determined by dividing the EC50 value of the serum against the respective mutant strain by the EC50 value of the serum against the wild-type strain. A heatmap was generated using Hem I, where the colour gradient from red to white corresponds to a multiplicative decrease in the ratio. The clade is derived from GISAID. (c) Sera from immunized subjects were assessed for neutralization against corresponding pseudoviruses by calculating the ID50 value, which indicates the serum dilution at which 50% neutralization is achieved. Neutralization differences for various pseudoviruses were calculated, with all values presented as means ± SD. (d) The X-axis lists each serum, and the Y-axis lists the different mutant pseudoviruses. The ratio was calculated in the same way as in Figure 3b. (e) Schematic diagram of the mouse immunization and challenge strategy.(f) ELISA method to assess the binding ability between serum and the corresponding protein.(g) Weight changes of mice over time, with the dashed line indicating a 25% weight loss.(h) Survival rate of mice over time.

To investigate whether single-point mutations affect the cross-neutralization ability, we collected serum samples after three rounds of immunization (Figure 3(a)). The neutralization activity of the serum was assessed against pseudoviruses (trimeric HA) packaged with G*ΔG-VSV from different strains (Supplementary Table 3). We found that BW06 (WT), V132I, D144N, and V132I + D144N exhibited relatively high cross- neutralization compared to other variants (Figure 3(b and c)).

Comparative analysis showed that only the sera elicited by antigens that lacked the T214A mutation retained high levels of cross-neutralization. We observed that the T214A mutation alone led to a significant reduction of cross-neutralization capacity, and a similarly dampened capacity was also observed in two other pseudoviruses carrying the T214A mutation combined with others. Interestingly, BW06, V132I, D144N, and V132I + D144N could cross- neutralization against all combinatorial variants including the T214A mutation, but the converse was not true, as neutralization levels significantly dropped in the presence of T214A (Figure 3(d)). Even when the residue at position 214 was not A or T, the results were similar (Figure 3(b), Supplementary Figure 2). Therefore, we speculated that the 214 T position of the RBS region is a crucial determinant of the cross-neutralization ability.

To investigate the differences in the in vivo protection levels between BW06 and BB08, we employed passive immunity using guinea pig serum and then challenged the mice with the virus (Figure 3(e)). Five hours after passive immunization, we collected blood from the inner canthus of the eye and verified the success of immunization via ELISA. If immunization was unsuccessful, the animals were excluded from subsequent challenge challenge statistics (n ≥ 4) (Figure 3(f)).

In the subsequent 5LD50 challenge with B/Austria/1359417/2021 virus, we observed that all mice in the BB08 and negative control groups had died by day 6. However, the mice treated with the positive control (B/Austria/1359417/2021 monovalent vaccine stock) BV21 and BW06 exhibited 60% and 75% survival rates, respectively, which delayed the progression of the disease (Figure 3(g and h)).

### Impact of the T214A mutation on broadly neutralizing antibodies (bnAbs)

**Figure 4.**
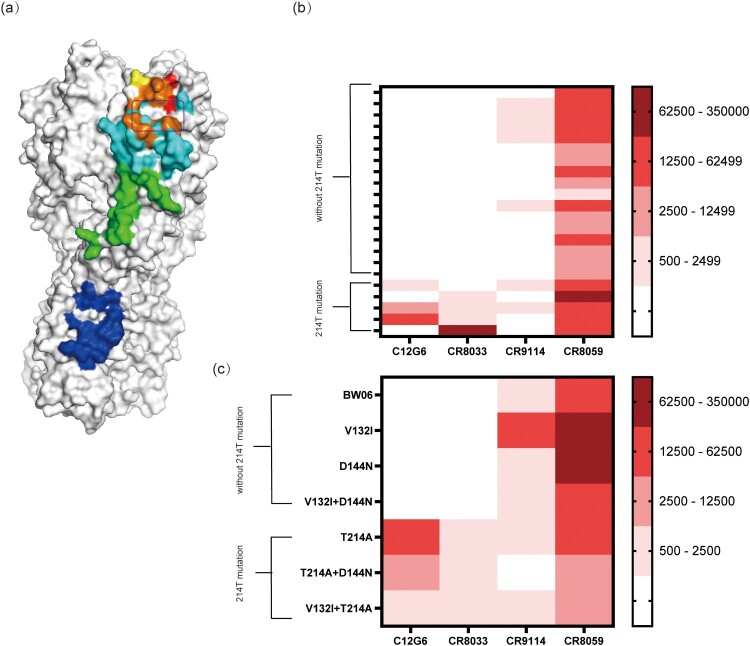
Study of Broadly Neutralizing Antibodies (bnAbs) Against Different Circulating Strains and Mutant Variants. (a) Schematic representation of the neutralizing epitopes recognized by the monoclonal antibodies C12G6 (light blue), CR8033 (red), CR8059 (green), and CR9114 (dark blue). The overlap between the epitopes recognized by C12G6 and CR8033 is indicated in orange, and residue 214 is highlighted in yellow. The RBS region is enclosed in a black box. (b) The neutralization levels of different broadly neutralizing antibodies against circulating strains from different years are illustrated based on the calculated ID50 values. White indicates no neutralization, while varying shades of red represent different levels of neutralization. (c) Neutralization levels of different bnAbs against variant antigens with point mutations.

To investigate the impact of the T214A mutation on broadly neutralizing monoclonal antibodies (bnAbs), we selected four antibodies: C12g6, CR8033, and CR8059, which target HA1, as well as CR9114, which targets HA2 [20,35] (Figure 4(a)). We found that two antibodies, C12G6 and CR8033, possessed poor broad neutralizing capacity against currently circulating strains (Figure 4(b)). Interestingly, after introducing the T214A mutation into BW06, the ID50 values for C12G6 and CR8033 antibodies significantly increased (Figure 4(b and c)). By contrast, the neutralizing capacities of CR9114 and CR8059 were not affected by mutations of residue 214. This suggests that changes of residue 214 are a key determinant influencing the recognition of the RBS region by broadly neutralizing antibodies.

### Epidemiological study of single-point mutations

**Figure 5.**
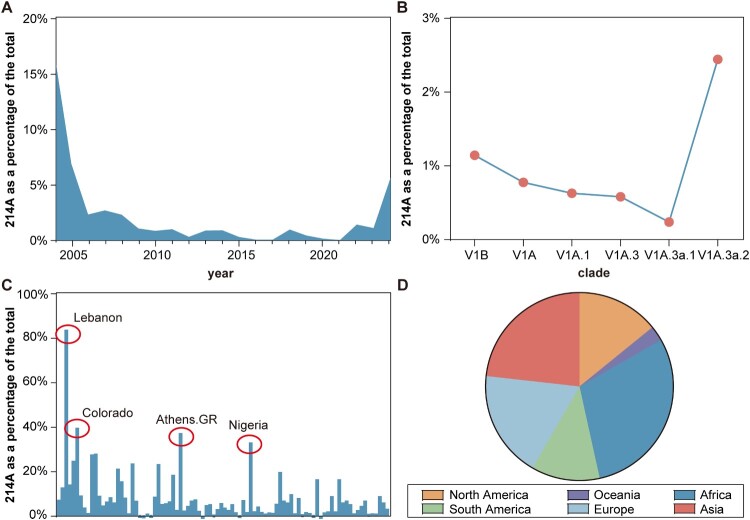
Overall Prevalence of the Hemagglutinin 214A Mutation (a) Annual proportions of the 214A mutation within the overall dataset. (b) Proportions of the 214A mutation across different clades within the overall dataset. To ensure data accuracy, branches with fewer than 100 viral sequences were excluded from the analysis. (c) Regional prevalence of the 214A mutation among total submissions within the V1A.3a.2 clade. (d) Prevalence of the 214A mutation across continents. To accurately reflect the data, the pie chart was created by calculating the weighted proportions as the sum of 214A mutation proportions submitted from each region within a continent, divided by the total submissions from all regions within that continent.

To investigate the prevalence of the 214A mutation across years and lineages, we downloaded all IBV HA sequences from the GISAID database. The HA sequences were aligned and screened to calculate the mutation rate of amino acid residue 214A among all discovered strains. Notably, the 214A mutation accounted for 15.8% of sequences in 2004, then decreased to 7% in 2005, and remained at a low percentage (below 3%) in among strains from subsequent years. However, in 2024, the frequency of the 214A mutation increased nearly fivefold compared to 2023, akin to the mutation rate observed in 2005 (Figure 5(a)). By analyzing the circulation of different sub-lineages, we found that the proportion of 214A increased progressively from 0% in V1A.3 to 3a.1 and 3a.2 (Figure 5(b)). We further analyzed submissions from various regions for the current prevalent clade V1A.3a.2. In Colorado (USA), Athens Georgia (USA), and Nigeria, the proportion of sequences with the 214A mutation approached 40% of all case submissions, while the strains reported in Lebanon shown a 214A mutation rate of over 80% (Figure 5(C)). By examining the prevalence of the 214A mutation across continents, we found that the occurrence of this mutation was slightly higher in Africa and Asia than in other regions (Figure 5(D)).

## Discussion

In addition to the outsized impact of the COVID-19 pandemic, influenza viruses were consistently considered as one of the major pathogens causing respiratory viral infections [1]. As of 2024, SARS-CoV-2 is no longer the dominant virus resulting in respiratory infections. There are four types of influenza viruses, with particular attention often being paid to influenza A. However, IBV also warrants attention as one of the most common pathogens that leads to severe respiratory infections [36]. The WHO prediction and introduction of influenza vaccines for each epidemic is based on the circulating strains, since constant genetic evolution of influenza leads to antigenic variation. These antigenic changes are primarily driven by amino acid substitutions near the receptor-binding domain of the HA head region [37–39]. In previous studies, we found that the neutralizing cross-reactivity among different influenza strains varied by a factor of 4–100 [4,40]. In our previous research on COVID-19, we discovered that certain single point mutations could significantly alter the infectivity and antigenicity of the virus, indicating that a tenfold reduction in the ID50 value could jeopardize the protective efficacy of existing vaccines [32,41,42]. In this study, we discovered that the antigenic change caused by the T214A mutation exceeded a tenfold alteration, which is rare, particularly since BW06 and BB08 belong to the same clade, yet a single T214A mutation was found to lead to significant changes in cross-neutralization activity. As of 2024, we have observed that although 214 T remains the dominant variant, the prevalence of 214A has been steadily increasing, with a fivefold rise compared to 2023. In fact, it has reached a peak in nearly 20 years, only surpassed by 2004, and this trend was also found in the circulating 3a.2 clade. Notably, GISAID data indicate that strains with the 214A mutation constitute over 80% of the V1A.3a.2 clade submissions from Lebanon. In Colorado, 110 out of 276 submitted sequences also contain the 214A mutation. Hence, this mutation has widespread prevalence across multiple continents and warrants attention. Notably, our research indicates no correlation between the T214A mutation and viral infectivity, suggesting that the prevalence of this mutation might impair vaccine protection.

Four main antigenic epitopes have been identified in IBV, including the 120, 150, 160 loop, and 190 helix [34]. Among them, the 190 helix serves as the receptor binding site (RBS) of hemagglutinin (HA) and is one of the most important epitopes. The amino acid residues at positions 194–196 are considered the most important hotspots within the 190 helix, as they represent potential glycosylation sites [43,44]. Previous studies have mainly focused on the addition or removal of HA glycosylation as a key mechanism for viral immune escape [34,45,46], but changes of glycosylation sites can also enhance the cross-protective ability of vaccines [16,22]. In our previous research, we demonstrated that the evaluation method using influenza pseudoviruses based on the vesicular stomatitis virus (VSV) backbone showed a good correlation with traditional hemagglutination inhibition and microneutralization assays [47,48]. In the current study, we found that variations at the 214 T position (consistent with position 196 of helix 190) significantly affected the cross-neutralization ability against pseudoviruses from different branches. This protection had a broad spectrum and was able to cross- neutralization against various mutations at this site, whereas the 214A variant only exhibits neutralizing capability for unchanged sites, while when this site mutates, the cross-neutralization ability is greatly diminished. and directly affected the development of heterologous viruses in the animals. Accordingly, we further hypothesized that this single point mutation may influence the “immunodominant” regions [49,50]. To validate this hypothesis, we predicted a glycosylation change at this site using the healthtech online server, which revealed that the 214A variant lacks a glycosylation site, and this absence enhances the “immunodominance” of this region, while 214 T covers this epitope through glycosylation, thereby expanding its reactivity. A similar phenomenon was observed in avian influenza, where single point mutations induced glycosylation changes affecting the cross-protection ability [51,52]. Our findings underscore the importance of the 214 T epitope in the selection of IBV vaccines.

The antigenic variation associated with the T214A mutation may impact the protective efficacy of vaccines. The circulating viruses in specific regions might mismatch with the recommend vaccine strains provided by the WHO, leading to inefficient protection and varying neutralization effects among vaccines. Since 2017, IBV carrying the 214 T mutation have become the predominant strains of selected vaccines, indicating a minimum impact on the neutralization efficacy against these strains.

Numerous studies are currently focusing on the development of broad-spectrum vaccines with enhanced cross- neutralization. Strengthening the cross- neutralization capacity of vaccines is crucial for addressing vaccine mismatch issues. Our findings highlight the importance of mutation sites with a strong impact on cross- neutralization, which require meticulous attention during vaccine strain selection. Many ongoing studies on broad-spectrum vaccines are focusing on targeting the conserved epitopes to enhance cross-protection, such as M2e, NA, NP, and HA2 [53–56]. Our study also suggests that single-point glycosylation mutations are also capable of providing cross-neutralization across IBV lineages or even groups, emphasizing a new insight for the redesigning of broad-spectrum vaccines.

Therapeutic monoclonal antibodies serve as a supplementary antiviral treatment, yet continuously face challenges from antigenic drift. Therefore, developing broadly neutralizing monoclonal antibodies can not only enhance their efficacy, but also provide a structural foundation for the development of vaccines with strong cross-neutralization activity. We validated the structures of four previously reported monoclonal antibodies against different circulating strains and mutant antigens [20,35]. We found that CR8059 exhibited neutralizing activity against all variants of IBV HA, likely because its epitope avoids the four major antigenic sites. Our research revealed that the T214A single-point mutation was able to restore neutralizing activity to the antibodies C12g6 and CR8033. This may be attributed to the additional interactions between the antibody's HCDR2 region and the HA 190 helix, which is crucial for receptor binding [27,57]. The T214A mutation alters the affinity between the 190 helix and the antibodies. Interestingly, C12g6 and CR8033 retain some neutralization capacity when residue 214 is mutated to N or I (Supplementary Figure 2).

This is corroborated by the results of the immunized sera. When the residue is 214 T, glycosylation at this position leads to the loss of effectiveness of antibodies that recognize the non-glycosylated epitope 214A.

The impact of single-point mutations is intriguing, and a recent study reported changes in the receptor binding preferences of HA due to single-point mutations [58]. Here, we demonstrated that single-point mutations can lead to significant changes of the neutralization capacity. Given the increasing prevalence of the 214A mutation in certain regions, it may pose a potential risk to public health. Therefore, the changes in animal protection levels associated with the T214A mutation warrant further attention in subsequent studies. Overall, these findings provide valuable insights for the design of next-generation broad-spectrum vaccines and antibodies.

## Supplementary Material

supplementary.docx
