# Supplementary material for: A single mutation at position 214 of influenza B hemagglutinin enhances cross-neutralization
Source: Emerg Microbes Infect. 2025 Feb 17;14(1):2467770. doi: 10.1080/22221751.2025.2467770 (PMC11849025; doi:10.1080/22221751.2025.2467770)
Supplement: supplementary.docx [file TEMI_A_2467770_SM3207.docx]

strain information Supplementary Table 1

| Isolate name | Isolate ID | Clade |
| --- | --- | --- |
| B/Wyoming/06/2014 | EPI_ISL_172672 | V1A |
| B/Brisbane/60/2008 | EPI_ISL_183972 | V1A |

The antibody sequences of CR8033, CR9114, and CR8059 are listed in Supplementary Table 2

| CR8033-VL | EIVLTQSPGTLSLSPGERATLSCRASQSVSSSYLAWYQQKPGQAPRLLIYGASTRATGIPARFSGSGSGTDFTLTISRLEPEDLAVYYCQQYGSSPWTFGQGTKVEIK |
| --- | --- |
| CR8033-VH | EVQLVESGGGLVQPGRSLRLSCAASGFSFDEYTMHWVRQAPGKGLEWVAGINWKGNFMGYADSVQGRFTISRDNGKNSLYLQMNSLRAEDTALYYCAKDRLESSAMDILEGGTFDIWGQGTMVTVSS |
| CR8059-VL | SYVLTQPPSASGTPGQRVTISCSGSSSNIGTNYVYWYQQFPGTAPKLLIYRSYQRPSGVPDRFSGSKSGSSASLAISGLQSEDEADYYCATWDDSLNGWVFGGGTKLTVL |
| CR8059-VH | EVQLVQSGAEVKKPGASVRVSCRASGYIFTESGITWVRQAPGQGLEWMGWISGYSGDTKYAQKLQGRVTMTKDTSTTTAYMELRSLRYDDTAVYYCARDVQYSGSYLGAYYFDYWSPGTLVTVSS |
| CR9114-VL | SYVLTQPPAVSGTPGQRVTISCSGSDSNIGRRSVNWYQQFPGTAPKLLIYSNDQRPSVVPDRFSGSKSGTSASLAISGLQSEDEAEYYCAAWDDSLKGAVFGGGTQLTVL |
| CR9114-VH | QVQLVQSGAEVKKPGSSVKVSCKSSGGTSNNYAISWVRQAPGQGLDWMGGISPIFGSTAYAQKFQGRVTISADIFSNTAYMELNSLTSEDTAVYFCARHGNYYYYSGMDVWGQGTTVTVSS |

Isolation strain information Supplementary Table 3

| Isolate ID | Submission Date | Clade |
| --- | --- | --- |
| EPI_ISL_9849525 | 2022 | V1A.3a.2 |
| EPI_ISL_9849542 | 2022 | V1A.3a.2 |
| EPI_ISL_14033365 | 2022 | V1A.3a.2 |
| EPI_ISL_410641 | 2019 | V1A.3a.2 |
| EPI_ISL_14033223 | 2022 | V1A.3a.2 |
| EPI_ISL_14033413 | 2022 | V1A.3a.2 |
| EPI_ISL_14033430 | 2022 | V1A.3a.2 |
| EPI_ISL_9849532 | 2022 | V1A.3a.2 |
| EPI_ISL_1519459 | 2021 | V1A.3a.2 |
| EPI_ISL_9849539 | 2022 | V1A.3a.1 |
| EPI_ISL_14033461 | 2022 | V1A.3a.1 |
| EPI_ISL_9849529 | 2022 | V1A.3a.1 |
| EPI_ISL_407532 | 2018 | V1A.3 |
| EPI_ISL_407231 | 2019 | V1A.3 |
| EPI_ISL_16884639 | 2019 | V1A.3 |
| EPI_ISL_293522 | 2017 | V1A.1 |
| EPI_ISL_14674 | 1982 | V1A |
| EPI_ISL_6617 | 1993 | V1A |
| EPI_ISL_219717 | 1985 | V1A |
| EPI_ISL_6819 | 1990 | V1A |

Supplementary Figure 1


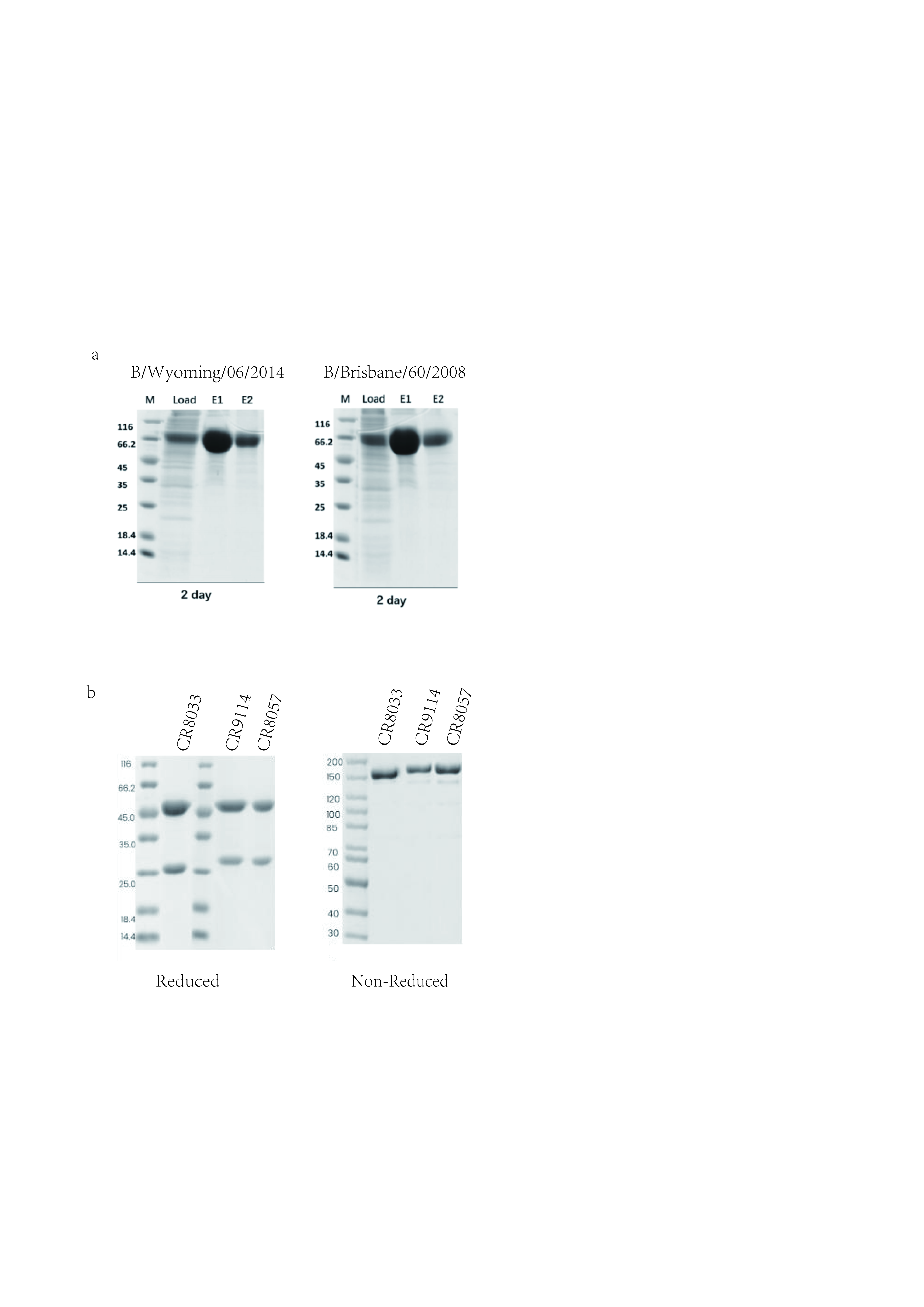


FigureS1:Protein and antibody purification WB results presentation (a) The protein purification results for B/Wyoming/06/2014 and B/Brisbane/60/2008 show that M represents the molecular weight of the protein, "load" refers to the sample loaded onto the chromatography column, and E1 and E2 are different concentrations of imidazole elution buffer. (b)The purification results for antibodies CR8033, CR9114, and CR8057.

Supplementary Figure 2


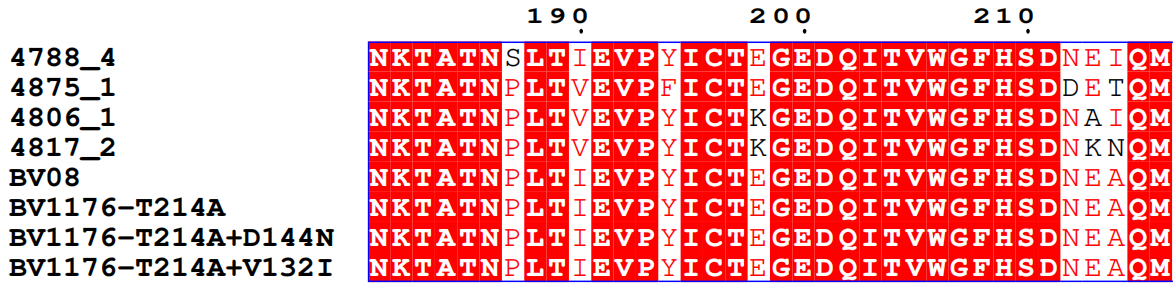


EPI_ISL_14674

EPI_ISL_219717

EPI_ISL_6819

EPI_ISL_169711

FigureS2:Strain information for variants at position 214 with I or N
